# Rehabilitation interventions targeting the activity and participation of patient with neuromuscular diseases: what do we know? A systematic review

**DOI:** 10.1055/s-0044-1779295

**Published:** 2024-02-23

**Authors:** Sionara Ferreira Silva, Hugo Leonardo de Magalhães, Franciele Angelo de Deus, Keysy Karoline Souza Andrade, Vanessa Pereira Lima, Thaís Peixoto Gaiad

**Affiliations:** 1 Universidade Federal dos Vales do Jequitinhonha e Mucuri, Faculdade de Ciências Biológicas e da Saúde, Departamento de Fisioterapia, Diamantina MG, Brazil.; 2 Universidade Federal dos Vales do Jequitinhonha e Mucuri, Programa de Pós-Graduação em Reabilitação e Performance Funcional, Diamantina MG, Brazil.

**Keywords:** Activities of Daily Living, Social Participation, Rehabilitation, International Classification of Functioning, Disability and Health, Neuromuscular Diseases, Atividades Cotidianas, Participação Social, Reabilitação, Classificação Internacional de Funcionalidade, Incapacidade e Saúde, Doenças Neuromusculares

## Abstract

**Background** To be objective and achievable, the rehabilitation goals must be focused on the functional expectations of patients with neuromuscular disease (NMD).

**Objective** Investigate rehabilitation programs that are able to modify the activity/participation of patients with NMD. Data search: Embase, BVS/Lilacs, Physiotherapy Evidence Database (PEDro), CINAHL/EBSCO, and Medline were searched in June 2021. It was last updated in March 2023.

**Methods** Randomized controlled trials investigating any rehabilitation therapy for patients with NMD with an outcome encompassing the activity/participation components of the International Classification of Functioning, Disability and Health (ICF) were included. Pharmacological therapy studies were excluded. The results were synthesized according to the ICF core sets for NMD. The methodological quality and level of evidence were assessed using PEDro criteria and Grading of Recommendations Assessment, Development, and Evaluation (GRADE). This systematic review followed the PRISMA 2020 guideline and was registered at PROSPERO (CRD42020209359).

**Results** Of a total of 1943 identified studies, 12 were included in this review with a methodological quality between regular and good. Light to moderate-intensity aerobic exercise was the most studied intervention. The mobility was assessed in all included studies.

**Conclusion** The variability of the types of NMD and the small sample size of the included studies demonstrates that there is very limited evidence of interventions focused on the activity/participation of individuals with NMD. Light to moderate-intensity aerobic exercise seems to improve the mobility, self-care, and social participation of patients with NMD, especially those with slow progression.

## Resumo

**Antecedentes** As metas de reabilitação devem ser focadas nas expectativas funcionais de pessoas com doenças neuromusculares (DNM) para que sejam objetivas e alcançáveis.

**Objetivo** Investigar programas de reabilitação capazes de modificar a atividade/participação de pessoas com DNM. Foi realizada busca nas bases de dados: Embase, BVS/Lilacs, Physiotherapy Evidence Database (PEDro), CINAHL/EBSCO e Medline em junho/2021. A última atualização foi realizada em março de 2023.

**Métodos** Foram incluídos estudos clínicos randomizados investigando qualquer terapia de reabilitação para pessoas com DNM com desfecho voltado para atividade/participação da Classificação Internacional de Funcionalidade e Saúde (CIF). Terapias farmacológicas foram excluídas. Os resultados foram sintetizados de acordo com os Core Sets da CIF para DNM. A qualidade metodológica e o nível de evidência foram avaliados usando os critérios PEDro e Grading of Recommendations Assessment, Development, and Evaluation (GRADE). Esta revisão sistemática foi registrada na PROSPERO (CRD42020209359).

**Resultados** De 1943 estudos identificados, 12 foram incluídos com uma qualidade metodológica entre regular e boa. O exercício aeróbio de intensidade leve a moderada foi a intervenção mais estudada. A mobilidade foi avaliada em todos os estudos incluídos.

**Conclusão** A variabilidade dos tipos de DNM e o baixo número amostral dos estudos incluídos contribuem para uma evidência muito limitada de intervenções focadas na atividade/participação de pessoas com DNM. O exercício aeróbio de baixa a moderada intensidade parece melhorar a mobilidade, autocuidado e participação de pessoas com DNM, especialmente para as DNM de progressão lenta.

## INTRODUCTION

Neuromuscular diseases (NMD) encompass acquired or inherited health conditions directly or indirectly affecting muscle function. These disorders result from deficiencies in various structures of the body, including muscles (e.g., Duchenne and Becker muscular dystrophy and myopathies), motoneurons (e.g., spinal amyotrophy and amyotrophic lateral sclerosis), peripheral nerves (e.g., Charcot-Marie-Tooth disease), or the neuromuscular junction (e.g., myasthenia gravis). Characterized by a progressive natural history, these conditions commonly manifest with weakness and fatigue and may also lead to spasms, muscle pain, dysphagia, dysarthria, and impairment of heart and respiratory functions. 1

The progressive condition and severity of NMD lead to limitations and restrictions in the performance of activities and participation, mainly in communication, mobility, self-care, household activities, interpersonal relationships, and community life. 2 From the perspective of individuals with NMD and their families, their priorities with respect to rehabilitation include mobility and transference, followed by self-care. 3 Nevertheless, the guidelines and research for the rehabilitation of patients with NMD mostly aim to assess the outcomes related to body structure and function. 4 5 6 7 Recent systematic reviews with conditions specific to the NMD group have been observed to report inconclusive or low evidence results for the role of rehabilitation directly on muscle function. 5 8 9 10 One study identified that strength training alone seems to have little or no effect in preventing disuse. 5 In this same vein, they reinforce that exercises in slowly progressive adult muscular dystrophies, such as facioscapulohumeral and myotonic dystrophy, should not be prescribed with the goal of restoring muscle strength. Their results indicate that physical therapists should focus on resistance training for the maintenance of mobility for slowly progressive muscular dystrophies and Charcot-Marie-Tooth disease (CMTD). 8

International Classification of Functioning (ICF) is a model for understanding the relationship that impairments in body structure and function, activity limitation, participation restrictions, and contextual factors (personal and environmental) have on the functioning of an individual from a biopsychosocial approach. 11 Individuals with Duchenne muscular dystrophy (DMD) have lower levels of social interactions, recreational activities, and skill development when older compared to younger patients. Besides this restriction impacting muscle disuse and physical deconditioning, this finding reinforces the importance of inclusion and the search for strategies that favour the performance of activities and social participation of these individuals. 12 The quality of life of individuals with myasthenia gravis (MG) or muscular dystrophy is not directly related to their muscle strength. 13 14 Individuals with myasthenia gravis perceive their quality of life to be negatively affected by difficulty in performing activities of daily living despite the use of medication to alleviate symptoms. For adults with muscular dystrophies, the quality of life is directly related to their perceived mental health, independence in performing activities, and management of fatigue and pain. The social participation of ambulant patients with amyotrophic lateral sclerosis is restricted mainly by physical limitations, followed by psychological factors. 15 Other factors that restrict the participation of these patient are lung capacity, functional mobility, fatigue, and feelings of helplessness.

Although the complaints of individuals with NMD are related to limitations of activity and participation restrictions, rehabilitation professionals experience difficulty in clinical decision-making since research directs the results to body structure and function. Knowing that the goals should be drawn together, centered on relevant complaints for the patient and whether they are achievable, 16 directing rehabilitation to body functions that will certainly be lost by the natural history of NMD makes it impossible to achieve the goals. To be objective and achievable, the rehabilitation goals must be focused on the functional expectations of these individuals, i.e., to improve the activities and participation through exercises, adaptations, and modifications of the environmental context by a multi-professional team. Thus, this study sought to identify the rehabilitation interventions that modify the activity and participation of patients with NMD.

## METHODS

This systematic review followed the Preferred Reporting Items for Systematic Reviews and Meta-Analyses (PRISMA 2020) and the AMSTAR 2 checklist for critically appraising systematic reviews of randomised controlled clinical trials. 17 18 This protocol was previously registered with PROSPERO (CRD42020209359).

### Study search and eligibility criteria

The search was conducted on EMBASE, BVS/Lilacs, Physiotherapy Evidence Database (PEDro), CINAHL/EBSCO, and MEDLINE databases in June 2021, updated in March 2023, without language or date restrictions. The reference lists of the retrieved studies were also searched to identify potential studies not otherwise identified in our searches. Randomized clinical trials (RCT), patient involving individuals with health conditions within the NMD category, were included. Any rehabilitation modality, exercise program, or therapy for patient with NMD of any age was considered for intervention, and interventions were from any area, such as physiotherapy, occupational therapy, physical education, speech therapy, or complementary therapy. The exclusion criteria comprised preclinical experimental studies, pharmacologic therapies, expert opinions, case reports, and abstract-only studies.

Studies with any domain related to the activity and participation in the ICF context as an outcome were considered. As comparators, any study, irrespective of the type of exercise or rehabilitation used, was eligible. Studies with no restriction on the date or language of publication were included.

To ensure saturation in the literature, the searches consisted of index terms (MeSh), free-text terms, and synonyms. The descriptors were organized into three groups:

- terms that describe the study population (patient with NMD) and its types.
- terms related to rehabilitation strategies.
- terms that refer to the ICF components. Supplementary Material 1 ( https://www.arquivosdeneuropsiquiatria.org/wp-content/uploads/2023/11/ANP-2023.0131-Supplementary-Material-1.docx ) presents the details of the search strategies for each searched database.

### Study selection

The steps of study selection, data extraction, methodological quality analysis, and certainty of the evidence of this review were developed by two reviewers independently (HLM and SFS), and a third reviewer was consulted in cases of disagreement (TPG). The studies obtained were exported to the Rayyan® program, and duplicate references were identified and removed. The reviewers screened the titles and abstracts. Duplicates were removed prior to selection. Subsequently, the full texts of potentially eligible papers were evaluated. All reviewers were physical therapists with expertise in NMD. The details of the excluded studies after the full-text review are listed in Supplementary Material 2 ( https://www.arquivosdeneuropsiquiatria.org/wp-content/uploads/2023/11/ANP-2023.0131-Supplementary-Material-2.docx ).

Studies with primary and/or secondary outcomes that addressed the activity and participation components of the ICF were selected. The reviewers analyzed each of the outcomes and the measurement instruments used for evaluation, adhering to the ICF core sets for NMD and following the division proposed by Whiteneck and Dijkers. 19 The NMD core sets list chapters 3 to 9 (d3: Communication, d4: Mobility, d5: Self-care, d6: Domestic life, d7: Interpersonal interactions and relationships, d8: Social, community, and civic life, and d9: Main areas of life) as significant items. The items in chapters 3 to 6 were considered activities and items in chapters 7 to 9 were considered participations. The definition of the outcome also adhered to the ICF manual categorizing part of the activity component if it aimed to intervene in a task or action performed by an individual. It was classified as part of the participation component when the intervention aimed to address issues of an individual's involvement in a real-life situation. 2 19

### Data extraction

The reviewers extracted the data using a standardized form:

- population (NMD type, sample size of NMD type, and age),
- intervention,
- comparator,
- activity/participation domains assessed, and
- outcome measure instruments.

### Methodological quality

The methodological quality of the studies meeting the inclusion criteria was evaluated using the PEDro criteria, yielding a score from 0 to 10. This scale comprises a total of 11 points, encompassing 10 items related to internal validity and 1 item related to external validity. It is important to note that, according to the scale's guidelines, the external validity item is not counted. The total score of 10 items is categorized into three groups: good methodological quality (7-10), regular (4-6), and poor (0-3) quality.

### Synthesis

The results were categorized according to the domains of activity and participation, aligning with the core sets for NMD ( Table 1 ). The determination of activity and participation domains was established through an examination of the outcomes and assessment instruments outlined in each study.

**Table 1:** Core Sets of Activity/Participation for patients with NMD

Domains	Definition according to ICF ^2^
**d3–Communication**	Communication through language, signs and symbols, including receiving and producing messages, maintaining conversation and using communication devices and techniques.
**d4–Mobility**	Movement when changing the position or location of the body, transporting, moving or handling objects from one place to another, walking, running or going up/down and using different forms of transport.
**d5–Self care**	Self-care such as washing and drying, taking care of the body and body parts, dressing, eating and drinking, and taking care of one's own health.
**d6 - Domestic life**	Shopping, storage, positioning of materials, food, clothing, planning and preparing meals, doing household chores, cleaning utensils and equipment, removing trash, helping family members.
**d7–Interpersonal interactions and relationships**	Respect, tolerate, express opinions, criticize appropriately, establish physical contact, interact according to social rules, and maintain social space. Establishing relationships when necessary with strangers, relating formally in certain environments, creating and developing friendly relationships with acquaintances, colleagues and family, relating intimately with others on an emotional, physical and sexual basis.
**d8 - Social, community and civic life**	Participate in community social life, participate in organizations, ceremonies, clubs. Participate in group recreational activities, go to the cinema, theater, play instruments, participate in religious or spiritual activities, enjoy nationally and internationally recognized human rights.
**d9 - Main areas of life**	Learning informal education with family members, such as crafts, leisure/fun, acquiring preschool and school education skills, performing lessons, fulfilling obligations and responsibilities, participating in professional tasks, acquiring and maintaining work.

### Overall certainty of evidence

Two authors independently assessed the certainty of the evidence for the primary outcomes using the Grading of Recommendation, Assessment, Development, and Evaluation (GRADE) framework methodology. Five GRADE domains, namely study limitations, consistency of effect, imprecision, indirectness, and publication bias, were analytically assessed. The final judgment on certainty was revised and downgraded by one or two levels as appropriate, reflecting the extent of bias in important quality domains. We used GRADEpro software® to present the study findings.

## RESULTS

**Figure 1:**
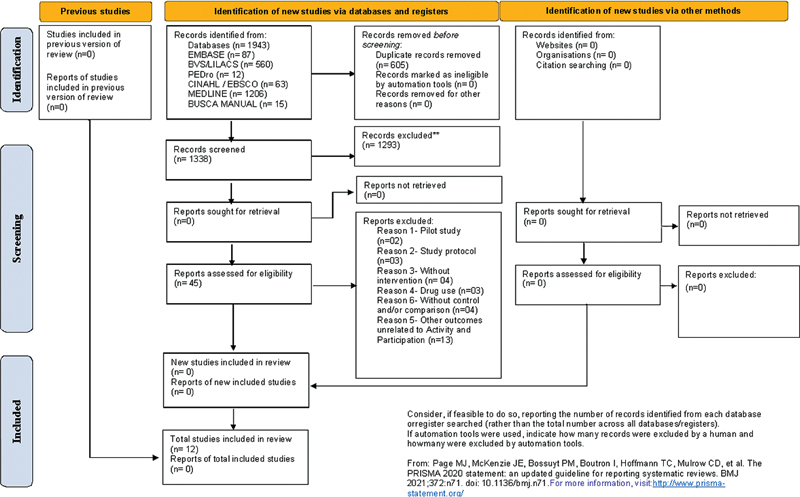
PRISMA 2020 flow diagram for updates systematic reviews which included searches of databases, registers and other sources.

The search strategy identified 1943 publications. After the title and abstract screening, a total of 45 publications were potentially eligible; after the full-text review, 12 publications were included. The details of the selection process are presented in the flowchart in Figure 1 . Table 2 describes the characteristics of the 12 included RCTs. The sample size of the studies ranged from 13 to 309 participants; the NMD was amyotrophic lateral sclerosis (ALS) in 5/12 studies, followed by facioscapulohumeral dystrophy (FSHD) in 4/12 studies, and hereditary motor and sensory neuropathy (HMSN) in 3/12 studies. The participants were of both sexes, and the studies were published between the years 1995 to 2021.

**Table 2:** Characteristics of the included studies (n = 12)

	NMD type	Sample size	Age (mean ± sd)	Severity	Intervention details	time and frequency of intervention	Outcomes				
							Mobility (d4)	Self care (d5)		Communication (d3)	Participation (d7, d8, d9)
Andersen *et al.* (2017) 20	FSHD1	(13) EG: N = 6 CG: N = 7	EG: 53 ± 15 (26–67) CG: 46 ± 9 (32–59)	FSHD score = 1–11	**EG:** HIT (cycle-ergometer) **CG:** Usual care	8 weeks (3 × 10-min)	6MWT 5STS IPAQ			
Dal Bello-Haas *et al. (* 2007) 21	ALS (ALSFRS early stage)	(27) EG = 13 CG = 14	not informed	not informed	**EG:** stretching exercise program (as CG) and an individualized UE and LE moderate-load intensity resistance exercise program **CG:** Usual care UE and LE stretching exercises	6 months	ALSFRS-R			
Lindeman *et al. (* 1995) 22	MD HMSN (types I or II)	(62) MyD = 33 HMSN = 29	MyD EG: 40 ± 11 CG: 37 ± 10 NHMS EG:35 ± 10 CG: 38 ± 11	not informed	**EG:** Strength training with weights of the proximal lower extremity muscles **CG:** nonexercising (CG were encouraged to refrain from training	3x week for 24 weeks	Time scored activities performed as fast as possible: ascending and descending stairs, rising from a chair, rising from supine on a physical exercise table and walking 50m 6MWT WOMAC functional part of the Western Ontario and McMaster Universit	SIP-68		SIP-68: Social participation
Nakajima et al. (2021) 23	NMD	(24) EG = 11 CG = 13	CG= 55,5 ± 7.2 EG = 56 ± 13.2	no rapid changes in gait symptoms for three months prior to the start of the trial	**GE:** Walking program with partial body support and exoeskeletal device for LE **GC:** Walking program with partial body support (30min)	13 weeks (9 sessions)	2MWT (gait distance) 10MWT (speed) Barthel index	Barthel index		
Okkersen *et al.* (2018) 24	MD1	(255) EG = 128 CG = 127	EG: 44,8 ± 11,7 CG: 46,4 ± 11,3		**EG:** Cognitive behavioural therapy intervention and usual care or aerobic exercise **CG:** Usual care	10 months (10–14 sessions)	DM1-Activ-C 6MWT MDHI	DM1-Activ-C	MDHI	DM1-Activ-C MDHI
Öksüz *et al.* (2011) 25	NMD	(60) EG = 30 CG = 30	EG: 37.5 ± 15.1 CG:38.7 ± 15.4	can stand in long leg braces but unable to walk even with assistance and who uses a wheelchair full time	**EG:** Client centred occupational therapy program and conventional therapeutic home exercise program by a physiotherapist **CG:** Conventional physiotherapeutic home exercise program by a physiotherapist	6 months	FIM DASH	FIM		COPM
Raglio *et al* . (2016) 26	ALS	(30) EG = 15 CG = 15	EG: 62.9 ± 9.83 CG: 65.1 ± 12.10	mild–moderate disability (ALS Functional Rating Scale- Revised ≤40)	**EG:** Active Music Therapy and usual care **CG:** Usual care	12 sessions, 3x/weeks (4 weeks)	ALSFRS-R	ALSFRS-R	MTRS	
Sherief *et al* . (2021) 27	DMD	(30) EG = 15 CG = 15	EG: 8.49 ± 0.83 CG: 8.34 ± 0.88	Able to walk alone level I and II of Ambulation function classification system for DMD (AFCSD)	**EG:** physical therapy program and aerobic exercise training by treadmill **CG:** physical therapy program and aerobic exercise training by bicycle ergometer	20 min, 3x/week, 3 months 20 min, 3x/week, 3 months	6MWT			
Van Groenestijn *et al.* (2019) 28	ALS	(57) EG = 27 CG = 30	EG: 60.9 ± 10.0 CG: 59.9 ± 10.7	life expectancy longer than 1 year; ability to walk with or without walking aid (≥10 min); and ability to cycle on a cycle ergometer (≥15 min)	**EG:** Aerobic cycling exercise program (50-75% HRR) and usual care **CG:** Usual care	16 weeks	ALSFRS-R TUG			SIP-68 IPAQ
Veenhuizen *et al.* (2019) 29	NMD	(53) EG = 29 CG = 24	EG: 52 (37-63) CG: 50 (41-60)	not informed	**EG:** Aerobic exercise training (50-70% HRR) and self-management strategies **CG:** Usual care	16 weeks 3x week of 30 min	6MWT			COPM
Voet *et al* . (2014) 30	FSHD1	(57) EG1 = 20 EG2 = 13 CG = 24	EG1: 59 (21–68) EG2:49 (24–69) CG: 52 (20–79)	Able to walk independently (ankle-foot orthoses and canes are accepted)	**EG1:** Aerobic cycling exercise training (50-65% HRR) **EG2:** Cognitive-behavioral therapy **CG:** Usual care	30 min, 3x/week, 16 weeks (GE1) minimum of 3 session (GE2)	6MWT CIS-activity	SIP-68		SIP-68
Zivi *et al.* (2018) 31	Peripheral neuropathies	(40) EG = 21 CG = 19	EG: 66.3 ± 13.0 CG: 71.8 ± 7.7	ability to maintain the upright position and to walk even if with assistance	**EG:** Gait and balance of aquatic physiotherapy **CG:** Gait and balance of on-land training	4 weeks of daily sessions of usual physiotherapy 3x week of specific treatment (aquatic vs. on-land)	DGI BBS FIM Functional Ambulation Classification			

Abbreviations: 10MWT (speed), 10 min walk test; 2MWT (gait distance), 2 min walk test; 5STS, 5-time sit-to-stand time; 6MWT, 6-min walk distance; ALS, amyotrophic lateral sclerosis; ALSFRS, Functional Rating Scale; BBS, Berg Balance Scale; CG, Control group; CIS-activity, Checklist Individual Strength; COPM, Canadian Occupational Performance Measure; DASH, Disabilities of the Arm Shoulder and Hand Questionnaire; DGI, Dynamic gait index; DM1-Activ-C, DM1-Activ-c scale; EG, Exercise group; FIM, Functional Independence Measure; FSHD1, facioscapulo-humeral muscular dystrophy type 1; HIT, high-intensity training; HMSN, hereditary motor and sensory neuropathy; HRR, heart rate reserve; IPAQ, International Physical Activity Questionnaire; LE, lower extremity; MD, myotonic dystrophy; MD1, myotonic dystrophy type 1; MDHI, Myotonic Dystrophy Health Index; MTRS, Music Therapy Rating Scale; NMD, neuromuscular diseases; sd, standart deviation; SIP-68, Sickness Impact Profile; SS, Sample size; TUG, Timed Up and Go; UE, upper extremity; WOMAC, functional subscale of the Western Ontario and McMaster University.

The interventions were aerobic exercise (7/12) and resistance exercise program (4/12), followed by cognitive behavioral therapy (2/12), active music therapy (1/12), aquatic exercise (1/12), and high-intensity training (1/12). The tested patients were compared either with control groups consisting of healthy individuals in four studies ( *referências* ) or with matched groups having the same NMD undergoing standard treatment in eight other studies ( *referências* ). The articles defined conventional/usual treatment as maintenance of rehabilitation with a multi-professional team or permission to perform exercises they had been performing before the beginning of the study. The period of interventions ranged from 4 to 24 weeks ( Table 2 ). The included studies had PEDro scale scores between 4 and 8, with each item detailed in Table 3 . Of these 12 studies, 6 studies had good methodological quality, 24 26 27 29 30 31 and 6 studies had regular quality. 20 21 22 23 25 28

**Table 3:** Methological quality of the included studies using the Physiotherapy Evidence Database (PEDro) scale (n = 12)

Study	1	2	3	4	5	6	7	8	9	10	Total
Andersen et al. (2017) 20	Y	N	Y	N	N	N	N	N	Y	Y	4/10
Dal Bello-Haas *et al.(* 2007) 21	Y	Y	N	N	N	Y	Y	Y	Y	N	6/10
Lindeman *et al. (* 1995) 22	Y	N	Y	N	N	Y	Y	N	Y	Y	6/10
Nakajima et al. (2021) 23	Y	N	Y	N	N	N	Y	N	Y	Y	5/10
Okkersen *et al.* (2018) 24	Y	N	Y	N	N	Y	Y	Y	Y	Y	7/10
Öksüz *et al.* (2011) 25	Y	Y	Y	N	N	N	N	N	Y	Y	5/10
Raglio *et al.* (2016) 26	Y	Y	Y	Y	N	N	Y	Y	Y	Y	8/10
Sherief et al. (2021)) 27	Y	Y	Y	N	N	Y	Y	N	Y	Y	7/10
Van Groenestijn *et al.* (2019) 28	Y	N	Y	N	N	Y	N	Y	Y	Y	6/10
Veenhuizen *et al.* (2019) 29	Y	Y	Y	N	N	Y	Y	Y	Y	Y	8/10
Voet *et al.* (2014) 30	Y	N	Y	N	N	Y	Y	Y	Y	Y	7/10
Zivi *et al.* (2018) 31	Y	Y	N	N	N	Y	Y	Y	Y	Y	7/10

Notes: 1. subjects were randomly allocated to groups (in a crossover study, subjects were randomly allocated an order in which treatments were received); 2. allocation was concealed; 3. the groups were similar at baseline regarding the most important prognostic indicators; 4. there was blinding of all subjects; 5. there was blinding of all therapists who administered the therapy; 6. there was blinding of all assessors who measured at least one key outcome; 7. measures of at least one key outcome were obtained from more than 85% of the subjects initially allocated to groups; 8. all subjects for whom outcome measures were available received the treatment or control condition as allocated or, where this was not the case, data for at least one key outcome was analysed by “intention to treat”; 9. the results of between-group statistical comparisons are reported for at least one key outcome; 10. the study provides both point measures and measures of variability for at least one key outcome.

### Domains of the activity and participation component and instruments

Four domains of activity and participation were identified in the included studies: mobility (d4), communication (d3), self-care (d5), and social participation (d7/d8). No RCTs were found with domestic life outcomes (d6) or for domains d7, d8, or d9 individually. The studies employed the term “social participation” to characterize situations investigating an individual's involvement in life situations. This was analyzed using instruments that assess the impact of the condition on various areas of life and the expectations regarding participation ( Table 1 ).

The functional tests or measurement instruments used to investigate mobility were generic: 6-minute walk test - 6MWT (6/12) and its variation to TC2M (1/12), Timed up and go - TUG (1/12), Dynamic Gait Index - DGI (1/12), Berg Balance Scale - BBS (1/12), Functional Independence Measure - FIM (2/12), Sit and Stand test (5 times) (1/12), 10-m walking speed - VM10M (1/12), Barthel index (1/12), and timed tests (1/12). The other instruments were specific for the condition studied, such as the ALSFRS-R for ALS or DM1-Activ-C for myotonic dystrophy type 1(MD-1).

To assess social participation, the generic Canadian Occupational Performance Measure (COPM) and the Sickness Impact Profile (SIP-68) were used for FSHD. The assessment of communication outcomes employed the Music Therapy Rating Scale (MTRS), a tool specifically designed to evaluate verbal and non-verbal communication. Regarding self-care assessment, the SIP-68 was the main instrument for the FSHD, myotonic dystrophy, and HMSN type I and II. For ALS, ALSFRS-R was used, and for MD1, the DM1-Activ-C Scale was applied.

### Mobility

Mobility was investigated in the 12 included articles. 20 21 22 23 24 25 26 27 28 29 31 Zivi et al. 29 investigated the effect of aquatic therapy based on gait/balance training in 40 individuals with peripheral neuropathies, comparing it with ground training. The aquatic therapy was subdivided into stages: relaxation exercises and breathing control, balance and postural control exercises, and gait exercises. Aquatic therapy for patients with peripheral neuropathies was as effective for mobility as gait training on land; however, the dynamic gait index (DGI) score was significantly higher in the aquatic therapy group.

Another study with 255 individuals with myotonic dystrophy type 1 used 10 to 14 sessions of cognitive behavioural therapy associated with aerobic exercises, such as running, cycling, or dancing, as intervention for 30 minutes 3 times a week. The control group underwent conventional multidisciplinary treatment in secondary care. The intervention was able to increase social participation and mobility in individuals, improving scores on the DM1-ActivC Scale and the 6MWT in 10 months. 24

Lindeman et al. 22 investigated lower limb strengthening for 24 weeks with 66 participants, being MD (n = 33) and NHMS type I and II (n = 29). Mobility was assessed using the timed test; 6MWT and WOMAC (Western Ontario and McMaster Universities Osteoarthritis Index) functional subscale. The intervention did not improve the mobility of patients with MD. However, it was observed to be effective in increasing the natural walking speed of participants with NHMS.

The mobility outcome was investigated in 30 individuals hospitalized with ALS, using active music therapy for 4 weeks. The evaluation was done through the ALSFRS-R, which is a specific scale for the evaluation of the clinical and functional evolution of ALS, which includes the motor function of the upper and lower extremities during the activities of daily living. The scale was applied at the beginning and end of the treatment and after 2 months, where the scale value was maintained. 26 Van Groenestijn et al. 28 investigated 57 ALS outpatients, comparing aerobic exercise using a cycle ergometer with usual care over a 16-week intervention. Mobility was assessed by ALSFRS-R (for global functioning) and TUG (for functional mobility), and the values were maintained between the intervention and control groups.

Bello-Haas et al. 21 investigated the effects of resistance exercises on function, fatigue, and quality of life through the ALSFRS in 27 ALS patients. The intervention group performed an individualized home exercise program consisting of daily stretching and resistance exercises of moderate load and intensity, 3 times a week for 6 months. There was a significant improvement in the ALSFRS scores compared to individuals receiving usual care 21_._

A sample of 57 individuals with FSHD received home-based aerobic exercise training on a bicycle ergometer 3 times per week (n = 20), or individual cognitive behavioural therapy (n = 13) with an emphasis on fatigue performing intervention 3 times per week. Control participants (n = 24) underwent usual care. Aerobic exercise and cognitive behavioural therapy were shown to be effective in increasing the physical activity level and distance travelled on the 6MWT. 30

In another study with patients with FSHD, 20 high-intensity exercise (HIT) on a cycle ergometer for 8 weeks was investigated. The subjects (n = 13) were evaluated with the 5x sit and stand test, IPAQ, and the 6MWT. The intervention group showed significant improvement in physical conditioning.

The effects of the energetic program on 53 patients with different types of NMD were investigated for 16 weeks. The program consists of different aerobic exercise modalities, such as rowing with an ergometer, walking on a treadmill, pedalling on a bicycle ergometer, associated with physical activity education sessions. The type of exercise was determined based on each participant's preference and motor skills. The training was divided into two parts; in the first nine weeks, the participants performed the training 2 times a week, and in the last 7 weeks, the participants performed the training 1 time a week under the supervision of the physiotherapist, with the intensity adjusted from 50 to 70% of maximum heart rate. During the first three weeks, the individuals received education about the principles of physical and aerobic training in NMD for 60 minutes. The mobility of the participants was assessed using the 6MWT. The energetic program improved the 6MWT distance walked and the social participation of patients with NMD when compared to the control group, which performed the usual care. 29

A sample of 60 adult individuals diagnosed with NMD investigated individual and client-centred occupational therapy over a 6-month period. The participating individuals who were treated with client-centred occupational therapy showed improvements in fatigue and functional independence as assessed by the FIM and upper limb mobility by the disabilities arm, shoulder, and hand (DASH). 25

A study with patients diagnosed with NMD who were unable to walk independently for 10 meters without the use of an assistive device assessed the gait function. Thirteen 40-minute sessions of partial weight-bearing walking associated with a lower limb exoskeleton were compared with a control group that performed only partial weight-bearing walking. The proposed exoskeleton involved connecting the human nervous system with a wearable cyborg hybrid assistive limb (HAL) robot. The intervention showed significant improvement in the distance walked (6MWT) compared to the control group. 23

A total of 30 boys with DMD who were already undergoing conventional physical therapy underwent cycle ergometer training vs. horizontal treadmill training in order to assess the functional capacity through the 6MWT. Both groups performed the intervention 3 times a week for 3 months, each session lasting 20 minutes. Patients who received treadmill training had their aerobic capacity and balance increased to a great extent compared to those who received cycle ergometer training. 27

### Social participation

Social participation was assessed using the COPM, SIP-68, Participation and Autonomy Impact Questionnaire (IPAC), DM1-Activ-C, and Myotonic Dystrophy Health Index (MDH) instruments in five studies. It was observed that lower limb strengthening of patients with MD and NHMS type I and II, 22 cognitive behavioral therapy 24 for patients with MD1, occupational therapy, 25 and aerobic exercise 28 29 for patients with NMD increased the participation of patients with MD, NHMS, ALS, FSHD, BMD, LGMD, myopathies, and neuropathies.

### Communication

Only two of the 12 included studies analyzed communication outcomes. Raglio et al. 26 investigated active music therapy (AMT) performed by a music therapist (12 sessions, 30 minutes, 3 times a week) during the hospitalization period of 30 individuals with ALS. The control group underwent the usual rehabilitation sessions of the service: physical therapy, speech therapy, occupational therapy, and psychological support. The communication was evaluated by MTRS for verbal and non-verbal communication. AMT did not show superior results than conventional treatment for the outcome communication and reported improvement in aspects of quality of life in the experimental group. 26 Okkersen et al. 24 investigated 255 individuals with myotonic dystrophy type I submitted to cognitive behavioral therapy and aerobic exercises. Positive results were evaluated using the MDHI instrument, indicating improvement in the communication outcome in the intervention group.

### Self-care

The outcome of self-care was evaluated through the SIP-68 in 57 individuals with FSHD1, submitted to aerobic exercises and cognitive behavioral therapy that was based on six modules directed to coping with the disease, dysfunctions related to fatigue, sleep, social support, and social interactions. The intervention was tailored to the specific needs of each participant, total number of sessions was based on the number of modules to be addressed, which were identified by the therapist through interviews and specific tests. All domains (except pain) were positively modified. 30

### Evidence overview

The quality of evidence of the RCTs included in this review was assessed using GRADE. Each of the endpoints was evaluated, and the synthesis of evidence was rated as very low due to inconsistency and indirect evidence (different types of interventions, low sample size, and heterogeneous population). All reasons for rating down are reported in Table 4 .

**Table 4:** Evidence quality for each outcome (GRADE)

	Sample size (Studies)	Evidence quality (GRADE)
**Mobility**	847 (12 RCT's)	very low ^2,3^
**Participation**	487 (05 RCT's)	very low ^1–3^
**Communication**	285 (02 RCT's)	very low ^1,2,3^
**Self care**	526 (05 RCT's)	very low ^2,3^
**Grade of evidence (GRADE)** 34 **High:** The authors have a lot of confidence that the true effect is similar to the estimated effect **Moderate:** The authors believe that the true effect is probably close to the estimated effect **Low:** The true effect might be markedly different from the estimated effect **Very low:** The true effect is probably markedly different from the estimated effect		

Notes: ^**1**^ Study limitations (risk of bias, such as lack of allocation concealment or blinding); ^**2**^ Imprecision (wide confidence intervals crossing a decision threshold); ^**3**^ Indirectness of evidence (differences in populations, interventions, comparators or outcomes); ^**4**^ Publication bias (missing evidence, typically from studies that show no effect).

## DISCUSSION

Our findings indicate that rehabilitation has an impact on activity/participation outcomes in individuals with NMD. However, these results should be interpreted with caution due to the limited evidence. While there are studies exploring outcomes aligned with the ICF Core Sets of Activity/Participation with moderate to good methodological quality, it is challenging to assert the effectiveness of the interventions in modifying these outcomes due to the small sample size and heterogeneity of NMD types.

Various interventions were investigated, the most frequent being aerobic exercise associated or not with cognitive behavioural therapy/health education for patients with Udd distal myopathy – tibial muscular dystrophy (UDM-TDM), MD1, and ALS. Some therapies, such as high-intensity exercise, are still controversial in patients with muscular dystrophy. However, Andersen et al. 20 concluded that HIT is safe, applicable, and tolerated by moderately affected patients with FSHD, a slowly progressing muscular dystrophy.

Considering the health conditions within the NMD group, a few diseases were investigated in the included RCTs: ALS, MD, FSHD, HMSN, DMD, MG, and spinal muscular atrophy (SMA). Thus, it is not possible to extrapolate the findings to the entire group of NMD. The studies did not include the nine proposed domains of activity and participation of the core sets for individuals with NMD. 2 Mobility, social participation, communication, and self-care were assessed in the studies. No studies were found that specifically evaluated the domains of home life (d6) and the domains of d7, d8, or d9 alone. In our analysis, the domains of personal interactions and social, community, and civic life were assessed by COPM and SIP-68 and termed as social participation by the authors. Researchers reported in a systematic review the limitation of studies focused on participation. However, most evidence with good quality and large samples studies medical intervention, e.g., drug treatment. 32 Cross-sectional studies investigating the participation of patients with NMD have been conducted with patients with Duchenne and Becker muscular dystrophy. The authors observed that participation is fundamental to favour social interaction; however, children with DMD prioritize activities that require less physical effort; thus, they demonstrate having reduced levels of recreational and social skills and activities. The non-participation of these individuals leads to a lower quality of life, affecting healthy aging and developing functional limitations. 12 33

Studies included in this systematic review present samples of varied ages, from children with DMD to adults with other types of NMD. Of these, only one RCT 27 investigated some intervention in the child population, thus showing the need for RCTs that have the ICF activity/participation component as an outcome for the child-youth population.

Aerobic exercise was the most studied intervention aiming at the mobility outcome. Among the modalities proposed are the use of the cycle ergometer, walking with partial weight support, and horizontal treadmill. The time of the interventions was short to medium term, from 04 to 24 weeks of intervention, with a single RCT studying the follow-up, which observed changes up to 11 months after the intervention. 29

The results of two other systematic reviews conducted by Gianola et al. 8 and Voet et al. 5 showed that besides knowing about the exercises proposed to individuals with NMD, it is important to know how to plan their execution 5 as well as the duration and intensity of the sessions. 8 The available evidence is still uncertain, suggesting that strengthening alone may have little or no effective response in the treatment. In general, the studies have small numbers of participants, and new studies with a larger population are needed since studies involving NMD have lower prevalences. 5 8 Although these two studies investigated the role of exercise in muscular diseases and had the body structure and function as the endpoint, the characteristic of the included RCTs confirms the reasons for our observation of low evidence. 5 8

The outcome of self-care was studied by five randomized clinical trials. 22 24 25 26 30 Among the interventions used, music therapy drew attention, which, in addition to promoting emotional well-being for the patient, reduced the perception of physical symptoms. In the group that received music therapy, the authors observed that individuals with ALS maintained the effects generated by the intervention for a longer period of time compared to the control group, besides presenting an improvement in the relationship and communication between the patient and the therapist. 26 These findings, although with few participants, demonstrate the usefulness of such a resource as a form of physical-functional treatment for these individuals with NMD, thus providing resources that favour the performance of physiotherapy, the comfort, and evolution of the patient, making possible the effectiveness of protocols, adherence to treatment, and the interest of the individual in achieving goals. 34 Moreover, when dealing with patients who have a progressive health condition, complementary resources that are accessible and easy to apply can compose the arsenal of rehabilitation strategies.

It was possible to identify that the measurement instruments used for the activity/participation outcomes were mostly generic. In order to target the assessment to the specificities of this NMD population, it is important to identify and use specific instruments.

To our knowledge, this was the first study to synthesize the interventions targeting the activity and participation of individuals with NMD and to analyze the quality and level of evidence of previous studies. However, some limitations of this study require consideration. The NMD group was not entirely included in this review, being restricted to some conditions, such as muscular dystrophies, ALS, and peripheral neuropathies. As in other studies with NMD, the sample size is small, with heterogeneity in the included population, two points that make RCTs difficult to perform.

In conclusion, given the heterogeneity of NMD and the small sample size of the included studies, the only low-evidence finding in this review is that aerobic training may improve the mobility of individuals with FSHD. Further randomized clinical trials with the NMD population are necessary to enhance decision-making on rehabilitation interventions that can modify activity/participation and address the functional priorities of this population.
