# Supplementary material for: Rehabilitation interventions targeting the activity and participation of patient with neuromuscular diseases: what do we know? A systematic review
Source: Arq Neuropsiquiatr. 2024 Feb 23;82(2):s00441779295. doi: 10.1055/s-0044-1779295 (PMC10890920; doi:10.1055/s-0044-1779295)
Supplement: Supplementary file 1 — Supplementary Material [file 10-1055-s-0044-1779295-s230131.pdf]

Supplementary Material 1

Search strategy

Database	
**EMBASE**	('neuromuscular diseases':ti,ab,kw OR 'neuromuscular disease':ti,ab,kw OR 'muscular diseases':ti,ab,kw OR 'muscular disease':ti,ab,kw OR myopathies:ti,ab,kw OR myopathy:ti,ab,kw OR 'muscle disorders':ti, ab,kw OR 'muscle disorder':ti,ab,kw OR 'myopathic conditions':ti,ab,kw OR 'myopathic condition':ti,ab, kw) AND ('exercise therapy':ti,ab,kw OR 'exercise therapies':ti,ab,kw OR 'rehabilitation exercise':ti,ab,kw OR 'physical medicine':ti,ab,kw) AND rehabilitation:ti,ab,kw
**BVS/LILACS**	(mh: "Doenças Neuromusculares" OR (neuromuscular diseases) OR (enfermedades neuromusculares) OR (doenças musculares) OR (muscular diseases) OR (enfermedades musculares) OR (doenças da junção neuromuscular) OR (neuromuscular junction diseases) OR (enfermedades de la unión neuromuscular) OR miopatias OR (distrofias musculares) OR (muscular dystrophies) OR mh: c05.651 OR mh: c10.668.491 OR mh: c10.668.758 OR mh: c10.668) AND NOT (mh: "Acidente Vascular Cerebral" OR stroke OR (accidente cerebrovascular) OR (doença de alzheimer) OR (alzheimer disease) OR (enfermedad de alzheimer) OR (doença pulmonar obstrutiva crônica) OR dpoc OR copd OR mh: c10.228.140.300.775 OR c14.907.253.855) AND (mh: "Reabilitação" OR (rehabilitation) OR (rehabilitación) OR (fisioterapia) OR (physical therapy specialty) OR (exercício físico) OR (exercise) OR (ejercicio físico) OR (terapia através da dança) OR (dance therapy) OR (terapia a traves de la danza) OR ioga OR yoga OR (tai ji) OR (tai chi) OR (tai chi chuan) OR mh: e02.831 OR mh: h02.010.625 OR mh: g11.427.410.698.277 OR mh: i03.350) AND (mh: "Classificação Internacional de Funcionalidade, Incapacidade e Saúde" OR (international classification of functioning, disability AND health) OR (clasificación internacional del funcionamiento, de la discapacidad y de la salud) OR (atividades cotidianas) OR (activities of daily living) OR (actividades cotidianas) OR (participação social) OR (social participation) OR (participación social) OR mh: i03.050.750 OR mh: l01.453.245.945.450 OR mh: sp5.006.057.193 OR e02.760.169.063.500.067 OR e02.831.067 OR i03.050 OR n02.421.784.110)
**PEDro**	'neuromuscular disease' 'muscular dystrophy'
**CINAHL – EBSCO**	(("Neuromuscular Diseases" OR "Muscular Diseases")) AND (("Exercise Therapy" OR "Rehabilitation" OR "Physical Therapy Modalities" OR "Physical and Rehabilitation Medicine")) AND (("International Classification of Functioning, Disability and Health" OR "Disability Evaluation" OR "Activities of Daily Living" OR "Social Participation")) NOT (("Radiculopathy" OR "Fibromyalgia" OR "Alzheimer Disease")))
**PUBMED/MEDLINE**	((((("Neuromuscular Diseases"[Mesh]) OR "Muscular Diseases")) AND ((("Exercise Therapy"[Mesh]) OR "Rehabilitation" OR "Physical Therapy Modalities" OR "Physical and Rehabilitation Medicine")))) AND ((("International Classification of Functioning, Disability and Health"[Mesh]) OR "Disability Evaluation" OR "Activities of Daily Living" OR "Social Participation")) NOT (((("Radiculopathy"[Mesh]) OR "Fibromyalgia"[Mesh]) OR "Alzheimer Disease"[Mesh]))

Supplementary Material 2

Excluded studies

Article	Excluded study	Title	Reason for exclusion
1	Voet, et al., (2010)	Effect of aerobic exercise training and cognitive behavioural therapy on reduction of chronic fatigue in patients with facio-scapulohumeral dystrophy: protocol of the FACTS-2- FSHD trial.	Study protocol
2	Kilmer, et al., (2005)	Impact of a home-based activity and dietary intervention in people with slowly progressive neuromuscular diseases	No control group
3	Birnbaum et al.,(2018)	The benefits and tolerance of exercise in myasthenia gravis (MGEX): study protocol for a randomised controlled trial.	Study protocol
4	Ayvat F, et al., (2019)	The International Classification of Functioning, Disability and Health-based factors related to physical activity level in adults with muscle diseases.	No intervention
5	Preisler, et al.,(2009)	Effect of aerobic training in patients with spinal and bulbar muscular atrophy (Kennedy disease).	No control group
6	El Mhandi, et al.,(2008)	Benefits of interval-training on fatigue and functional capacities in Charcot-Marie-Tooth disease.	No control group
7	Sveen, et al.,(2008)	Endurance training improves fitness and strength in patients with Becker muscular dystrophy.	Wrong study design
8	Chetlin, et al., (2004)	Resistance training effectiveness in patients with Charcot-Marie-Tooth disease: recommendations for exercise prescription.	Drug use
9	Orngreen, et al., (2015)	Aerobic training in patients with myotonic dystrophy type 1.	No control group
10	Ahlström, et al., (2006)	A comprehensive rehabilitation programme tailored to the needs of adults with muscular dystrophy.	Does not describe the intervention
11	Peeters, et al., (2019)	Don’t forget the trunk in Duchenne muscular dystrophy patients: more muscle weakness and compensation than expected.	Wrong study design
12	Menotti, et al., (2014)	Amount and intensity of daily living activities in Charcot-Marie-Tooth 1A patients.	No intervention
13	Lancioni, et al.,(2017)	A basic technology-aided programme for leisure and communication of persons with advanced amyotrophic lateral sclerosis: performance and social rating.	Drug use
14	Duong, et al., (2021)	Understanding the relationship between the 32-item motor function measure and daily activities from an individual with spinal muscular atrophy and their caregivers’	No intervention
15	Hind, et al., (2017)	Aquatic therapy for children with Duchenne muscular dystrophy: a pilot feasibility randomised controlled trial and mixed-methods process evaluation [with consumer summary]	Pilot study
16	Olsen, et al.,(2005)	Aerobic training improves exercise performance in facioscapulohumeral muscular dystrophy.	Wrong study design
17	Burns, et al.,(2019)	Safety and efficacy of progressive resistance exercise for Charcot-Marie-Tooth disease in children: a randomised, double-blind, sham-controlled trial	Different outcome
18	Veenhuizen, et al.,(2021)	Mixed methods evaluation of a self-management group programme for patients with neuromuscular disease and chronic fatigue.	Wrong study design
19	Hsu, et al.,(2019)	A Touch-Observation and Task-Based Mirror Therapy Protocol to Improve Sensorimotor Control and Functional Capability of Hands for Patients With Peripheral Nerve Injury.	Study protocol
20	Misra, etal.,(2021)	Rest or 30-Min Walk as Exercise Intervention (RESTOREX) in Myasthenia Gravis: A Randomized Controlled Trial	Drug use
